# Algorithm of chest wall keloid treatment

**DOI:** 10.1097/MD.0000000000004684

**Published:** 2016-09-02

**Authors:** Xiao Long, Mingzi Zhang, Yang Wang, Ru Zhao, Youbin Wang, Xiaojun Wang

**Affiliations:** Department of Plastic Surgery, Peking Union Medical College Hospital, Beijing, China.

**Keywords:** algorithm, chest wall, keloid, surgery

## Abstract

Keloids are common in the Asian population. Multiple or huge keloids can appear on the chest wall because of its tendency to develop acne, sebaceous cyst, etc. It is difficult to find an ideal treatment for keloids in this area due to the limit of local soft tissues and higher recurrence rate. This study aims at establishing an individualized protocol that could be easily applied according to the size and number of chest wall keloids.

A total of 445 patients received various methods (4 protocols) of treatment in our department from September 2006 to September 2012 according to the size and number of their chest wall keloids. All of the patients received adjuvant radiotherapy in our hospital. Patient and Observer Scar Assessment Scale (POSAS) was used to assess the treatment effect by both doctors and patients. With mean follow-up time of 13 months (range: 6–18 months), 362 patients participated in the assessment of POSAS with doctors.

Both the doctors and the patients themselves used POSAS to evaluate the treatment effect. The recurrence rate was 0.83%. There was an obvious significant difference (*P* < 0.001) between the before-surgery score and the after-surgery score from both doctors and patients, indicating that both doctors and patients were satisfied with the treatment effect.

Our preliminary clinical result indicates that good clinical results could be achieved by choosing the proper method in this algorithm for Chinese patients with chest wall keloids. This algorithm could play a guiding role for surgeons when dealing with chest wall keloid treatment.

## 1 Introduction

**Figure 1:**
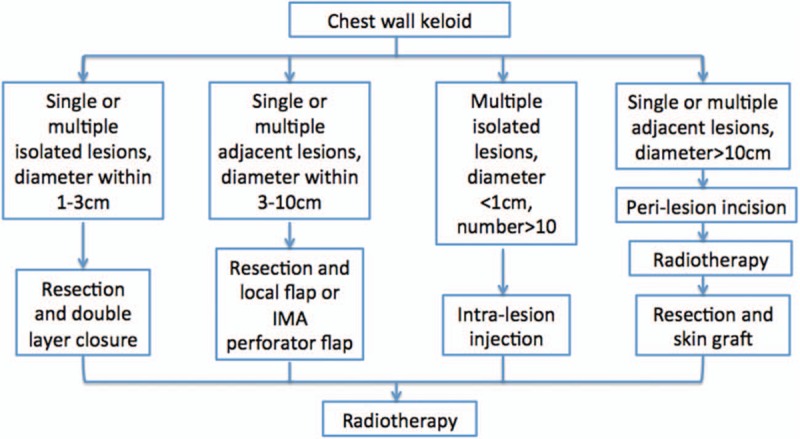
Algorithm of chest wall keloid treatment. According to the size, number, and location of chest wall keloid, 4 protocols are available for choosing in this algorithm. IMA = internal mammary artery.

Keloid has been one of the most challenging clinical problems for many years. Most physicians recommended that management of the keloid depends on the patient's individual circumstances and evidence-based findings.^[1,2]^ Despite the wide range of therapeutic methods available, keloid scars typically still have a high rate of recurrence. It is reported that nearly half of the keloids occurred at the anterior chest wall area.^[2]^ In addition, patients also care about the symptom relief, skin color, thickness, and pliability. In order to treat the chest wall keloid more effectively, we established an algorithm (Fig. 1) from which an individualized protocol could be easily selected according to the size and number of chest wall keloids.

## 2 Methods

### 2.1 Patient selection

Four hundred forty-five patients (242 males and 203 females; protocol 1: 177 males and 138 females; protocol 2: 29 males and 28 females; protocol 3: 10 males and 10 females; protocol 4: 26 males and 27 females) received various methods of treatment in our department from September 2006 to September 2012, with age ranges from 19 to 58 years (average age: 32.20 ± 6.39 years; average male age: 32.52 ± 6.54 years, average female age: 31.81 ± 6.21 years). Patients selected in this study had no comorbidity or systematic diseases, or took any drug that might affect the development of keloid or the study results. This clinical study protocol was reviewed and approved by the Ethical Committee of Peking Union Medical College Hospital. Informed consent for surgery and photo publication was provided by all of the patients before surgery.

### 2.2 Constitution of algorithm

According to the size, number, and location of the chest wall keloid, 4 protocols are available for choosing in this algorithm. There is no significant difference of age and sex between patients (*P* > 0.05), and no intervention was performed in each protocol. The keloid diagnosis was made and confirmed by a plastic surgeon and pathological examination from Department of Pathology, Peking Union Medical College Hospital. Radiotherapy was also necessary in treating keloid and was given in Department of Radiotherapy, Peking Union Medical College Hospital, as well.

#### 2.2.1 Protocol 1: keloid resection and direct closure with intradermal double layer continuous suture technique

Three hundred fifteen patients enrolled in this protocol. For the single or multiple isolated lesions with the diameter within the range 1 to 3 cm, we chose to perform keloid resection and direct closure. In order to prevent wound tension, double layer continuous intradermal suture was applied. Keloid resection was performed under local anesthesia and the superficial fascia was closed by nonabsorbable interrupted sutures. Then the first layer of continuous intradermal suture was placed with 3-0 monofilament nylon. The suture began from 1 side of the wound, extended at the deep dermal layer, and ended on the other side of the wound. Each end of the thread was fixed on the skin with a piece of silicone tube. The deep dermis would be approximated after this procedure. Another layer of continuous intradermal suture was placed in the superficial dermis layer with 3-0 monofilament nylon. The wound was then fixed with strips and covered with sterile gauze. Radiation therapy was given on the first and seventh days postoperation. Sutures can be removed easily at the 14th to 21st days postoperation.

#### 2.2.2 Protocol 2: keloid resection with local flap or internal mammary artery perforator flap

Fifty-seven patients enrolled in this protocol. For chest wall lesions with diameter >3 cm, local flap or internal mammary artery (IMA) perforator flap was used to cover the defect and avoid wound dehiscence. For lesions <5 × 5 cm, randomized local flap could be obtained and the donor site was closed primarily. For a larger keloid with diameter measuring 5 to 10 cm, IMA perforator flap will be a better option. The flap could be designed into a pedicled propeller style according to the direction of different IMA perforators that are detected by handheld Doppler. Keloid resection and defect coverage could be finished simultaneously, and radiotherapy was given to both the donor site and the recipient site at the first and seventh days postoperation.

#### 2.2.3 Protocol 3: perilesion incision + first session of radiotherapy + skin graft + second session of radiotherapy

Twenty patients enrolled in this protocol. For huge keloids, >10 × 10 cm, skin graft is the only option after keloid resection. Tie-over on the grafted skin will interrupt the radiotherapy; thus, we chose to perform an incision around the lesion and radiotherapy was given for the first time on the first day postoperation. Keloid resection and skin graft were performed immediately after the first radiotherapy. Ten to 14 days later, after the tie-over was removed, radiotherapy was given a second time.

#### 2.2.4 Protocol 4: keloid core resection and radiotherapy

Fifty-three patients enrolled in this protocol. For multiple lesions <1 × 1 cm, keloid core resection and radiotherapy twice (total 18 Gy) were given according to the international recommendations on scar management.^[1]^ Usually we design a 1- to 2-mm incision at 1 side of the keloid, and then dissect the dermis layer from the other side. Keloid core was removed and 5-0 absorbable sutures were used to close the space. Then the incision was closed by interrupted sutures with 6-0 nylon.

### 2.3 Radiotherapy

A total of 18 Gy over 2 sessions in the early postoperative period was given with Primus M Siemens linear accelerator, German, as recommended.^[3]^

### 2.4 Treatment effect assessment

The mean follow-up time was 13 months (range: 6–18 months). The Patient and Observer Scar Assessment Scale (POSAS) was used to evaluate the treatment effect, which is reported to be a reliable and valid method of assessing keloid scars in a clinical context.^[4]^ In this study, POSAS was given to both doctors and patients, to avoid potential sources of bias, once before surgery and once on patients’ return visit. The treatment effect was assessed by scores of POSAS. The higher the score, the poor the effect is. The total score for doctors’ scale and patients’ scale is 50 and 60, respectively.

### 2.5 Statistical analysis

All the data presented in this study are mean ± standard deviation. Statistical significance was determined via *t* test. Statistical significance was set at *P* < 0.05. All analyses were conducted by using SPSS 22.0, America.

## 3 Results

In mean follow-up time of 13 months, 18.65% (83 out of 445, 61 in protocol 1, 10 in protocol 2, 3 in protocol 3, 9 in protocol 4) of total patients were lost to follow-up. Both the doctors and the patients (362 in total) themselves used POSAS to evaluate treatment effect twice (there is no significant difference of age and sex between patients who participated in this study and who were lost to follow-up [*P* > 0.05]). Two of the 315 patients who followed protocol 1 suffered delayed wound healing. One of the 57 patients who followed protocol 2 suffered partial flap loss. No serious or obvious complications happened to patients who followed protocol 3 and protocol 4. Among the 362 patients, 0.83% of patients (3 patients) experienced obvious recurrence. The POSAS scores given by doctors and patients before and after surgery in each protocol are shown in Table 1. After surgery, the scores given by both doctors and patients were remarkably lower compared with the before-surgery scores. There was an obvious significant difference between the before-surgery score and the after-surgery score from both doctors and patients, indicating that both doctors and patients were satisfied with the treatment effect.

**Table 1:**
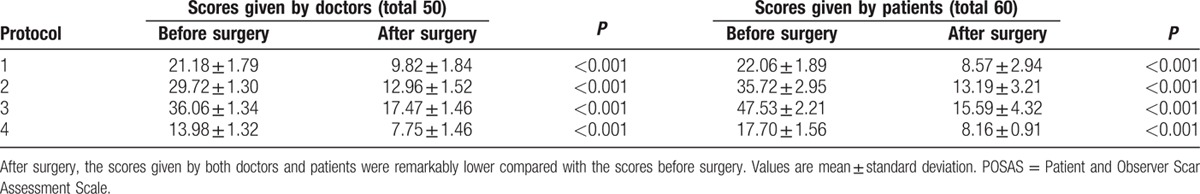
The POSAS scores of doctors and patients in each protocol.

### 3.1 Typical cases

#### 3.1.1 Cases 1 and 2

**Figure 2:**
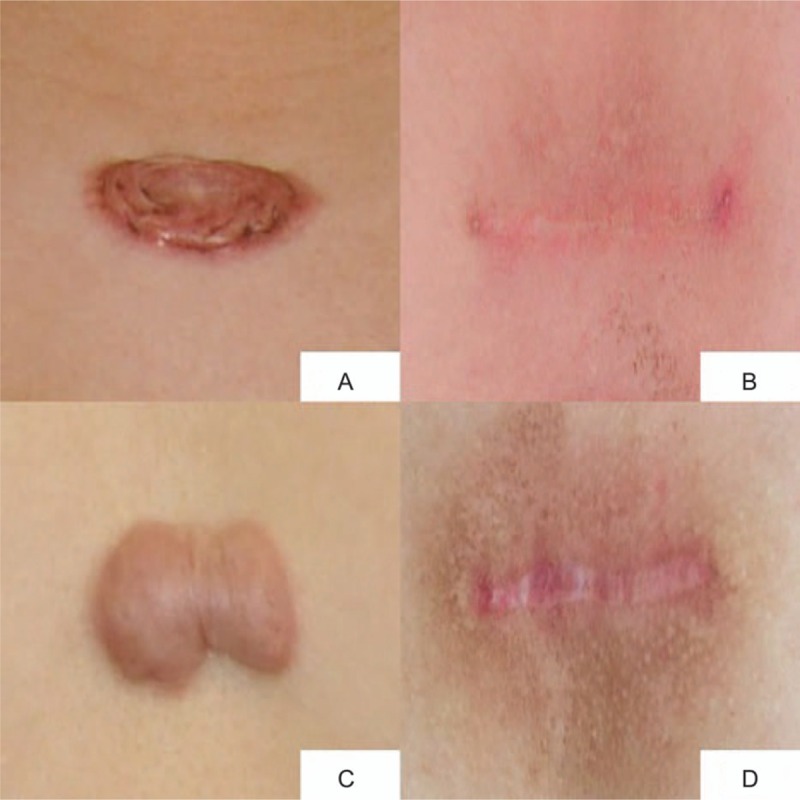
Clinical results of two cases of dual layer continuous suture of the keloid resection defect. (A and C) Preoperation keloid with the size of (3–5) × (1.5–2) cm each. (B and D) One year postoperation. Patients were satisfied with the treatment effect.

Two young patients suffered presternal keloids, with the size of (3–5) × (1.5–2) cm each. Keloid resection was performed and double layer continuous suture was applied followed by radiotherapy. Figure 2A and C shows the preoperation appearance, while Fig. 2B and D shows the postoperation result after 1 year.

#### 3.1.2 Case 3

**Figure 3:**
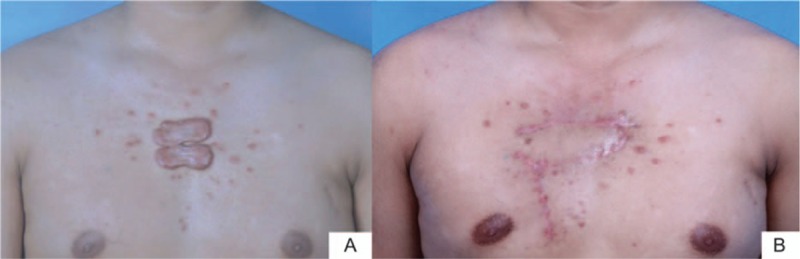
Chest wall keloid resection and local flap coverage of the defect. (A) Two adjacent lesions on the presternal area with the total size of 6 × 8 cm. Keloid resection and local flap was performed followed by sequential radiotherapy. (B) One year post–local flap transfer and radiotherapy. The patient was satisfied with the healing scars.

A 28-year-old male patient suffered multiple adjacent chest wall keloids with the total size of 6 × 8 cm. Keloid resection and local flap was performed followed by sequential radiotherapy. Figure 3A and B shows the preoperation and postoperation result after 1 year.

#### 3.1.3 Case 4

**Figure 4:**
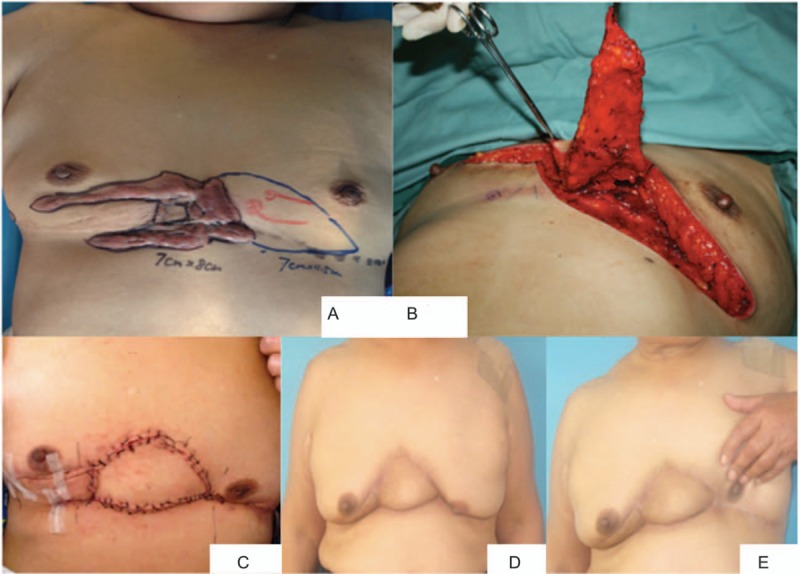
Clinical result of internal mammary artery perforator flap according to the lesion and the location of the perforator. (A) Preoperation chest wall keloid with the total size of 7 × 8 cm. (B) The location of the perforator. The defect after keloid resection was repaired by IMA perforator flap with the size of 7 × 4.5 cm. (C) Two weeks after operation. The incision healed well with no infection and exudation. (D and E) One year postoperation and radiotherapy with no recurrence. The patient was satisfied with the result. IMA = internal mammary artery.

A 58-year-old female patient had a relatively big keloid with the total size of 7 × 8 cm in the chest wall. The defect after keloid resection was repaired by IMA perforator flap. Figure 4A shows the preoperation view, Fig. 4B shows the IMA perforator flap, Fig. 4C shows the appearance on the first postoperative day, and Fig. 4D and E shows the 12 months’ result postoperation.

#### 3.1.4 Case 5

**Figure 5:**
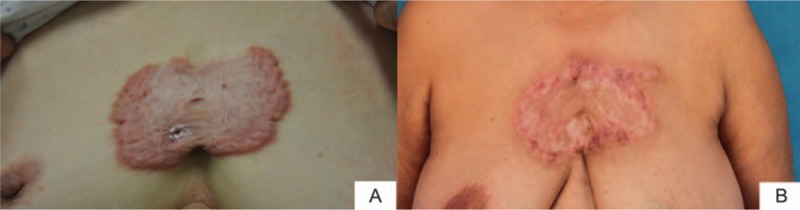
Incision around the keloid, radiotherapy, keloid resection, and skin graft with another session of radiotherapy. (A) Chest wall keloid with the size of 15 × 10 cm preoperation. (B) Twelve months post–keloid resection and skin graft. The patient was satisfied with the effect.

A 54-year-old female patient suffered from a chest wall keloid with the size of 15 × 10 cm. A full-thickness incision around the lesion was made followed by the first time of radiotherapy on day 1 postoperation, and at the same day keloid resection and skin graft was performed followed by the second session of radiotherapy 14 days later. Figure 5A and B shows the preoperation and postoperation views with 12-month follow-up.

## 4 Discussion

Multiple authors recommended individualized treatment for keloid in literature.^[5–8]^ A chest wall keloid has a higher recurrence rate due to multiple reasons.^[9–13]^ The proper treatment should be free of tension with a smooth healing process. Also, the method should ensure the postoperation radiotherapy.

### 4.1 Double layer intradermal continuous suture

An effective way of preventing keloid recurrence is tension release. Three-layer suture in the subdermal, dermal, and epidermal layers was first used by Hyakusoku and Ogawa.^[14]^ To avoid suture marks, skin adhesive was used and the running sutures were buried in epidermal layer. Ogawa et al emphasized the importance of subcutaneous/fascia tensile reduction sutures in their report.^[2]^ All of these measures were effective in preventing keloid recurrences, but dermal layer closure is also important. Interrupted intradermal suture with interrupted epidermal suture and epidermal buried running suture are common traditional dermal closure methods.^[15]^ The dermal sutures usually were left in the tissue, which increased the possibility of foreign body reaction and scar formation. Connective tissue capsules formed around threads were nonabsorbable, which might lead to cicatrization such as foreign body granuloma or keloids.^[16,17]^ In this case, absorbable sutures are preferred by many surgeons, but the possibilities of scar widening should be kept in mind. Verhaegen^[18]^ demonstrated that wide scar or wound dehiscence may become the consequence if the suture strength cannot equal the wound-shearing forces before the scar maturation.

Based on these principles, double layer continuous intradermal suture was designed. Besides superficial fascia tension reduction suture, the wound tension was effectively reduced by the deep layer continuous intradermal suture. Fourteen to 21 days after operation, this layer of suture can be easily removed. In our clinical work, no suture thread is left and foreign body reaction is finally prevented. Scar after operation is less obvious than that after traditional suture methods.

### 4.2 Application of local flap

Flaps have multiple benefits in treating a chest wall keloid. With sufficient skin and soft tissues, a flap could maximally reduce the skin tension. It was reported that the width and surface area of the perforator flaps had expanded 120% to 130% at 7 months after they were used for broad scar contractures.^[18]^ Thus, for the chest wall wound post–keloid resection, when it is difficult to close it primarily, local flaps are the best option. It has long been recognized that the IMA perforators will support the skin of the anterior chest wall; therefore, there are multiple choices when choosing IMA perforator flap to cover the defect. The width of the flap should be designed within the range of primary closure of the donor site. A freestyle or pedicled propeller flap based on the fourth or fifth IMA perforator could also be used in such circumstances with the most invisible scar within the inframammary fold.^[19]^

### 4.3 Application of skin graft

The main problem associated with skin grafting is the keloid recurrence at the margins of the skin graft.^[2]^ Therefore, based on the thesis that a keloid is the result of abnormal wound healing and it is critical to prevent its formation at the early stage of injury, we designed the protocol as follows: making an incision at the keloid margin, first session of radiotherapy, keloid resection and skin graft, and second session of radiotherapy. By making the incision around the keloid, a new wound is formatted and healing process is restarted. Then radiotherapy at the early stage postoperation could help to prevent the recurrence at the margin area. After this, keloid resection and skin graft was performed. At the 10th to 14th days postoperation, second session of radiotherapy was given after the tie-over being removed, which is still within the early postoperation period as recommended.^[1]^ Our preliminary study on 445 patients with huge keloids who were treated by this protocol using skin grafting and radiotherapy in our department has shown that all cases had uneventful postoperative courses and keloid recurrence was not observed. A longer follow-up period is needed to confirm these outcomes, but at present they are encouraging.

### 4.4 Keloid core removal technique

It is essential to put the incision in the normal tissue around the keloid during keloid core removal. One- to 2-mm normal epidermis could ensure the normal healing after radiotherapy. During the dissection, a thin layer of dermis should remain to avoid tissue necrosis. Usually we observe the edge of the incision for 5 to 10 minutes to make sure that the blood supply is adequate. Once the incision edge turns pale, it should be trimmed before wound closure.

However, the population in each protocol is not specifically symmetrical. More patients were involved in protocol 1; on the other hand, other protocols had few patients. This is 1 possible bias in this study. In this case, other protocols should receive more patients for a better analysis and more specific conclusion.

## 5 Conclusion

Keloid remains a frustrating disease for both doctors and patients. Our preliminary clinical result indicates that good clinical results could be achieved by choosing the proper method in this algorithm for Chinese patients with chest wall keloids. This algorithm could play a guiding role for surgeons when dealing with chest wall keloid treatment.
